# Person-centred care on the move – an interview study with programme directors in Swedish higher education

**DOI:** 10.1186/s12909-022-03657-4

**Published:** 2022-08-01

**Authors:** I. Björkman, C. Feldthusen, E. Forsgren, A. Jonnergård, I. Lindström Kjellberg, C. Wallengren Gustafsson, M. Lundberg

**Affiliations:** 1 University of Gothenburg Centre for Person-Centred Care (GPCC), Sahlgrenska Academy, University of Gothenburg, Gothenburg, Sweden; 2 Institute of Health and Care Sciences, Sahlgrenska Academy, University of Gothenburg, P.O. Box 457, 40530 Gothenburg, Sweden; 3 Department of Health Promoting Science, Sophiahemmet University, Stockholm, Sweden

**Keywords:** Person-centred care, Higher education, Local course syllabus change, Health care professionals, Interview study

## Abstract

**Background:**

There is an increasing trend towards person-centred care (PCC) worldwide, suggesting that PCC should be mastered by future health care professionals. This study aims to explore programme directors’ views on facilitators and barriers to implementing PCC in four of the largest national study programmes in Sweden training future health care professionals.

**Methods:**

A qualitative design was applied and interviews were conducted with 19 programme directors of Swedish national study programmes in medicine, nursing, occupational therapy and physiotherapy. The interviews were analysed using qualitative content analysis. Themes were sorted according to the Consolidated Framework for Implementation Research (CFIR) in an abductive approach. COREQ guidelines were applied.

**Results:**

The overarching theme, as interpreted from the programme directors’ experiences, was ‘Person-centred care is on the move at different paces.’ The theme relates to the domains identified by the CFIR as outer setting, innovation, inner setting and process. PCC was understood as something familiar but yet new, and the higher education institutions were in a state of understanding and adapting PCC to their own contexts. The movement in the outer setting consists of numerous stakeholders advocating for increased patient influence, which has stirred a movement in the inner setting where the higher educational institutions are trying to accommodate these new demands. Different meanings and values are ascribed to PCC, and the concept is thus also ‘on the move’, being adapted to traditions at each educational setting.

**Conclusion:**

Implementation of PCC in Swedish higher education is ongoing but fragmented and driven by individuals with a specific interest. There is uncertainty and ambiguity around the meaning and value of PCC and how to implement it. More knowledge is needed about the core of PCC as a subject for teaching and learning and also didactic strategies suitable to support students in becoming person-centred practitioners.

**Supplementary Information:**

The online version contains supplementary material available at 10.1186/s12909-022-03657-4.

## Background

There is an increasing trend towards person-centred care (PCC) throughout the world, advocated by different stakeholders, including the World Health Organization (WHO), patients, relatives and health care professionals (HCPs) [1–4]. Different definitions and understandings of PCC exist, with the common denominator being mutual respect and co-creation of health and care where the patient is an active partner in and not a passive recipient of care [4, 5]. Studies have found that PCC improves patients’ health [6], staff’s work satisfaction and health [7], and that it is financially viable [8, 9]. Such increased quality of care and cost-effectiveness are driving the implementation of PCC in clinical practice which has now gained momentum [10–12].

PCC could thus be expected to be a prominent feature at educational institutions responsible for educating future HCPs [1, 13, 14]. In an Australian context, a complete transformation of curriculum for nursing was done to reflect person-centredness [15, 16] by using a model for PCC practice by McCormack & McCance [17]. In Europe, higher education is driven by the Bologna process, which focuses on general national learning objectives, and freedom is given to universities and colleges to design content and implement local programme syllabuses and local course syllabuses [18, 19]. A previous study from our group exploring the PCC content in national study programmes, found that content related to PCC (including related concepts such as patient-centred consultation or client-centred practice), was limited, a finding in line with a study from Canada [20, 21].

For the purpose of the present study PCC is seen as a ‘health innovation’, which, according to the WHO, includes ‘new or improved’ health policies, practices, services and delivery methods that aim to improve the quality of health care. Innovation in an educational setting can involve the content of the curriculum, teaching methods or design [22] and aims to improve students’ learning. Implementation, on the other hand, is described as systematically ‘introducing’ something to become a natural element in ordinary activities, and several theoretical frameworks can be used [23, 24]. The implementation of innovations in educational settings is often challenging due to several barriers such as lack of time, high staff turnover and inefficient leadership [22, 25]. To gain a deeper understanding of why PCC is only partly implemented, although urged by numerous stakeholders, we wanted to explore the programme directors’ experiences of what inhibits and facilitates such implementation.

## Methods

### Aim

The aim was to explore facilitators and barriers to implementation of PCC in Swedish national study programmes in medicine, nursing, occupational therapy and physiotherapy.

### Study design and participants

The study had qualitative design and was performed in Sweden as part of a larger project aiming to map and explore content related to PCC in Swedish national study programmes training future HCPs. In spring 2020, 10,895 students were admitted to programmes in four areas of study, namely, in medicine, nursing, occupational therapy and physiotherapy. The participants in the present study were programme directors responsible for such programmes. COREQ guidelines were applied [26].

All programme directors, *n* = 48, in national study programmes (first cycle) in medicine, nursing, occupational therapy and physiotherapy, were invited by e-mail, with an aim to provide a variation regarding programme and geographical location. Non-responses were followed up with email and/or phone calls. Nineteen programme directors agreed to participate, among whom thirteen programmes had content related to PCC care in local programme syllabuses and/or course syllabuses. See Table 1 for an overview of sample characteristics.

**Table 1:** Participants’ characteristics

Study programmes	
Medicine	*N* = 4 (7^a^)
Nursing	*N* = 7 (25^a^)
Occupational therapy	*N* = 5 (8^a^)
Physiotherapy	*N* = 3 (8^a^)
Type of institution	
University	*N* = 11
University college	*N* = 8
Geographical location	
North	*N* = 4
West	*N* = 4
Middle	*N* = 3
East	*N* = 5
South	*N* = 3

^a^Total number of Swedish institutions providing the programme

Individual in-depth interviews were conducted via telephone or Zoom videoconferencing software by IB, IKL and AJ, between January and March 2021. The interviews were semi-structured and an interview guide was used (see Additional file 1). The interviews lasted for 22–67 min. Three pilot interviews were performed to check the validity of the interview guide and the interview technique. The interview guide remained unchanged, but the interview technique was revised so that the interviewer was more open to following the informants’ nuances and different understandings of the phenomenon. The interviews began with the same introduction, the interviewer briefly presenting herself and the project, asking for background information and then starting with the same introductory question as stated in the interview guide. Subsequent questions were not asked in any particular order, but as they came up naturally during the interview. Continual reflections and discussions were held between all authors regarding the interviews’ unfolding to attain sufficient breadth and depth.

### Data analysis

The analyses were performed during the period from April 2021 to November 2021. The interviews were audio recorded and transcribed verbatim by professional transcribers. All files (sound and text) were stored according to the relevant ethical codes and to Swedish law. The transcripts resulted in 323 pages of double-spaced text. The transcripts of the interviews were analysed in an iterative process, through an abductive content analysis inspired by Graneheim and Lundman [27, 28]. An abductive approach implies a movement between inductive and deductive analysis where a theoretical framework was applied in the process to gain a deeper understanding [29].

Initially, the interviews were listened to in full and transcripts were read individually to get a sense of the whole. Meaning units related to the research question were extracted from the transcripts and entered into Microsoft 365 Excel software, and then condensed and coded. These initial steps were performed by IB, AJ and CF and validated with all authors several times to attain consensus and striving for fidelity to the participants’ descriptions without losing the underlying meaning. The analysis thus started with an inductive approach in which coding categories were derived directly from the text data. To interpret the data in relation to implementation more clearly, we decided to structure the categories using the Consolidated Framework for Implementation Research (CFIR) [30], which identifies different domains relevant to implementation of innovations, namely, innovation, inner setting, outer setting, process and individuals. The CFIR was chosen to support the overall structure of the data from an implementation perspective in line with the aim of the study. Through an abductive endeavour, categories within each domain were compared for differences and interpreted as tentative themes [29].

The themes were based on the latent content of the totality of the material, interpreted by the researchers. This stage was refined in several meetings. Again, through a process of reflection and discussion, all authors then agreed on themes. Credibility was ensured by ML giving methodological support in selecting the most suitable meaning units as well as checking the coding procedure along the process. The codes were compared with each other and sorted into categories. In addition, peer debriefing with members in our research group was used to strengthen the credibility of the results. See Table 2 for examples from the analytical process.

**Table 2:** Examples from the analytical process

Meaning unit	Category	Domain CFIR	Subtheme	Theme
It felt like that was where we had to go. And it’s quite obvious now if you think about these government reports on primary care reforms, when everyone starts talking about this.	National governing documents and guidelines	Outer Setting	Multiple stakeholders pushing for PCC	**An OUTER SETTING characterized by a societal movement towards increased patient influence**
We are traditionally pretty good at examining knowledge. We are kind of good at skills. But how do we handle this with attitude and how do we examine it in a legally secure way? It’s not easy.	Examination	Process	PCC involves complex teaching and learning	**A PROCESS for implementation that is ongoing and fragmented**
The meaning of person-centred care is nothing new at all, but it is supported by law, professional ethical codes and key concepts of physiotherapy	PCC is nothing new	Innovation	PCC as something familiar yet new	**Forming an understanding of the INNOVATION and adapting it to each programme**

### Ethical considerations

This study was conducted according to the Helsinki Declaration [31]. Interviewees were informed about the aim of the study and their right to withdraw at any time. According to the Swedish Ethical Review Authority, no ethical approval was needed (Reference number 2020-05677).

## Results

**Fig. 1:**
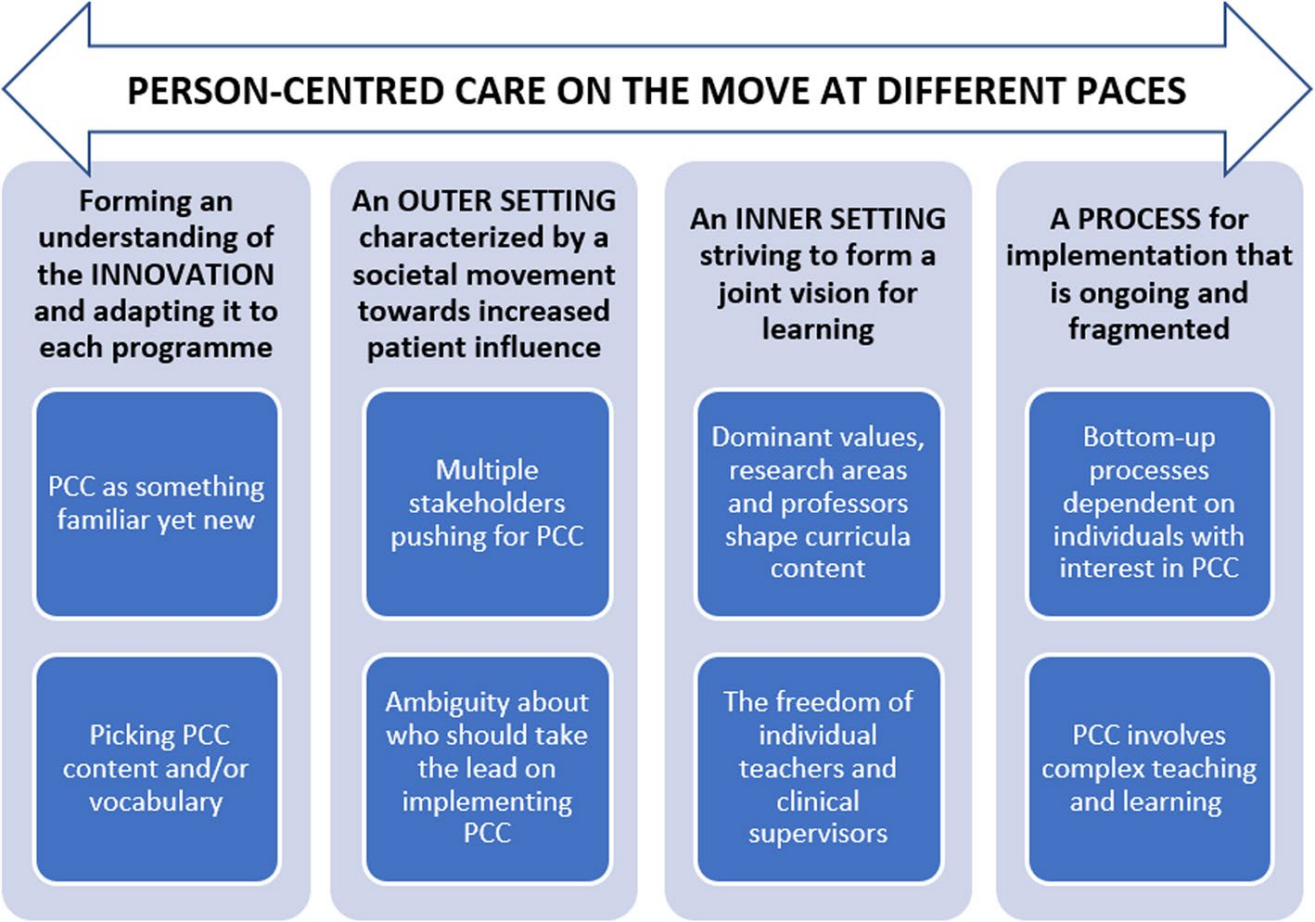
Overview of subthemes and themes in relation to overarching theme and CFIR

The analytical process resulted in subtheme and themes related to four domains of the CFIR: innovation, outer setting, inner setting and process. The overarching theme, as interpreted from the programme directors’ experiences, was ‘PCC is on the move at different paces’. This movement applies to the outer setting where increased patient influence is advocated by numerous stakeholders, which stirs activities in the inner setting where the educational institutions try to accommodate those stakeholders. At the same time there is no consensus on the value and meaning of PCC; thus, the concept itself is ‘moving’ as programme directors and the institutions they represent are in a process of grasping and adapting PCC to their respective contexts. Thus, PCC moves at different levels, as the educational institutions are in the midst of a change process that is inevitable but at the same time ambiguous. An overview of the themes and subthemes can be found in Fig. 1.

### Forming an understanding of the innovation and adapting it to each programme

PCC was understood as something familiar but yet new, and several different understandings existed among the programme directors. The various understandings of PCC ranged from it being a new vocabulary to be applied to an already existing content to an ethical framework comprising a radically new view on patient–HCP relations and patient participation.

“And as I understand person-centred care, it really takes it a step further. That you don’t view the patient really as just a patient, but you see the person, you see the actual person that you have in front of you and their background, prerequisites and wishes” (Programme director of medical programme).

Some participants expressed that their knowledge was limited and that they needed to learn more about PCC. The different understandings of PCC voiced by programme directors pointed toward a non-consensus and ambiguity around the concept. They had different understandings of what PCC entails and thus how much, if anything, they actually needed to implement. Furthermore, they did not agree on the value of PCC in comparison to other aligned perspectives, where some found it absolutely crucial for future HCPs and others felt it could be included in the programme as a minor theme. A value of PCC identified by several programme directors was of forming a joint language and being an umbrella term for all professions to gather under to work in teams. The ambiguity and non-consensus about PCC can be interpreted as reflecting a high complexity of the innovation and thus a barrier for implementation; on the other hand, it could also reflect a high degree of adaptability, which is then a facilitator.

All the represented institutions were in a state of understanding and adapting PCC to their own different contexts. Thus, picking PCC content and/or vocabulary was considered important, as opposed to ‘buy[ing] the whole package’ represented by different models and frameworks depicted in the literature. The vocabulary associated with PCC, such as labelling the individual in need of healthcare services a *person*, *patient* or *client* was described as value laden. At the same time choice of words was understood as unimportant, since it is the content that matters, and all the different words can be seen as synonyms and used interchangeably.

“I can start by saying that we do not have it [PCC] as a subject as such. So I would not say that we are discussing person-centred care with that terminology. But on the other hand, we try to person-centre the treatment in … by using, for example, a consultation model where we start with the patient’s part”. (Programme director of medical programme).

The tension between viewing PCC as content or as vocabulary meant that teaching on ethics, law or communication skills in general was sometimes viewed as synonymous with teaching of PCC. Thus, several programme directors stated that they did teach PCC without mentioning either the word person or the word centred. This could be interpreted as a barrier for implementation or as only a partial implementation having been conducted when the content was there but under a different vocabulary.

### An outer setting characterized by a societal movement towards increased patient influence

PCC was considered by the programme directors to be part of a strong movement towards increased patient influence in the surrounding society, and pushed by multiple stakeholders such as politicians, patient organizations and professional unions. Many programme directors described an ‘inevitability’ of PCC and an openness towards the influence of these different stakeholders. The movement was experienced as growing stronger over the years and was understood as aiming to equalize asymmetries in both knowledge and power between patients and HCPs.

“there are the laws and legislations that we have but also, really, the research that moves in that direction… that it is the individual who has the right to decide and choose and has the right to say no as well. So, I think it’s at that stage now in society, for us in Sweden” (Programme director of occupational therapy programme).

When PCC was found congruent with ethical codes for the professions, advocated by professional associations and supported by policy documents, this facilitated implementation. The programme directors compared their local course syllabuses to other equivalent programmes and strived to be at the forefront and educate for the future. Thus, if one higher educational institution implemented PCC, it facilitated implementation at other locations. Nevertheless, the programme directors also considered whether ‘all the talk about PCC’ in the last couple of years could be perceived as it being only a buzzword lacking true meaning. Understanding PCC as a passing trend, that is, something temporary, was thus an identified barrier.

The informants agreed that PCC is something national study programmes cannot neglect, but at the same time there was ambiguity about who should take the lead in implementing it, the higher educational institutions or the health care system. On the one hand, the programme directors argued, the students can act as pioneers and drive the transition towards PCC, but on the other hand, they were aware that a gap between on campus and clinical training was confusing to students.

“Knowledge sticks with the student differently if they don’t experience a gap between what is said in teaching and what is actually done in practice. If you want to introduce person-centred care, then it is important that it is incorporated as … that it is integrated [into clinical training] is very important. For students, it is difficult to deal with the gap. It can be very frustrating and difficult to get acceptance as new knowledge when students see that there is a gap.” (Programme director of physiotherapy programme).

Having clinical training at settings where PCC was practised and students experiencing clinical role models were identified as facilitators for implementing PCC at the corresponding educational institutions, while a fear of confusing students and training them for something they might not be able to practice was a barrier.

### An inner setting striving to form a joint vision for learning

Based on the programme directors’ views, forming a joint vision for learning could be difficult, since there are many influences within the inner setting on what perspectives and content should be prioritized. Curricula were described as ‘full’ already, and introducing new content meant that something else must be excluded. Content of local course syllabuses was considered strongly influenced by dominant values, such as profile areas of the institution or the whole university/college as well as traditions. These traditions could apply to content of teaching as well as ways of teaching, that is, pedagogical and didactic approaches. Leading figures and advocates for dominant values and perspectives are professors, and the perspectives they carry are passed on to other teachers and students. The research focus of the higher education institution is also influential on local course syllabuses, where those who do research in the areas where PCC is actualized are pushing for implementation in education.

“And I think that [what is to be included in curricula] also comes with the Professors – who are Professors. I think they are pretty influential in this. Actually… because they are also subject representatives. Yes, and points out the direction we should take.” (Programme director of nursing programme).

How the vision for learning was then realized was dependent on individual teachers and, most importantly, the placement supervisors, who form strong role models for students. Programme directors stated that the individual teachers and course coordinators have a lot of freedom in how and what they teach. Thus, a teacher or course coordinator can include and teach PCC even if it’s not in a local programme syllabus; however, even if PCC is in local course syllabuses, teachers might not carry out the learning activity the way it was intended. To include students’ clinical training under a shared vision was described as very challenging, since it was carried out at a number of sites and contexts. The programme directors expressed that they had minimal power over the content of clinical training, which is dependent on the placement supervisors’ interests and that they form the strongest role models for students. Consequently, what is taught at campus does not have the same impact on students as the clinical culture.

“But one would like the educational programme to be a driving force in this, that the young, the new would … But the problem is that young people often become very conservative when they experience a very strong [clinical] culture, and they do.” (Programme director of medical programme).

Developed communication channels between the university and the clinical settings were described as very important, but these are complicated by the turnover of employees. One factor described as a facilitator was adjunct supervisors being employed by both the university and the clinic. The individual teachers, course coordinators and placement supervisors are all potential facilitators for implementation if these are positive towards PCC; otherwise, they can act as barriers.

### A process of implementation that is ongoing and fragmented

The process of implementation was mostly carried forward through informal collegial discussions, even though some programme directors did describe having seminars for the staff to discuss and learn about PCC. They described a bottom-up process of implementation where individuals with interest in PCC were drivers, and no real formal leaders of the process were appointed. Programme directors described being at different stages of, as well as differing in their goals for, the implementation, where some strived to base their entire programmes on PCC so that everything was ‘permeated’ by it. Others described it as being included in certain courses or found existing content equivalent to PCC and thus found no need to change curricula or teaching. The programme directors had difficulties defining a specific starting point for implementation, but described various processes where the perspective gained ground step by step and found its way into teaching and local curricula. This was contrasted with having it included in the national study programme where a more planned course of action would take place with specific resources allocating time and educational efforts of teachers.

“You really need time and space to stop and think, and think through, to grasp the different nuances. And there is not always time for that.” (Programme director of nursing programme).

Individuals with a specific interest in PCC, as well as invited guests such as researchers in the field, were described as facilitators for implementation. The fact that PCC is not mentioned in the national study programme was described as a barrier for implementation, even if a majority felt it could be derived from the national study programme. Another identified barrier was lack of time.

PCC involves complex teaching and learning, and there were many ideas of how teaching and learning PCC was achieved, even though not all programme directors were directly involved in teaching. There was a consensus that PCC (or what was seen as equivalent content) should be introduced early, in the first semester, so that it becomes self-evident and comes naturally to the students. The importance of reflection was stressed, which also involves self-reflection, and it is argued that practising PCC presumes some personal maturity and growth. It is important that teaching starts from real-world experiences, such as cases from clinical training. An emotional engagement is needed from students that can be triggered, for example, by patient narratives.

It was stated that PCC is not declarative knowledge but needs to be developed over time and that the new students tend to apply it in a technical way. Those programme directors who had more extensive experience of teaching PCC shared that it requires a student-centred approach where the teachers need to be mindful of the students’ personhood and resources. Apart from the complexity of teaching and learning the subject, it was noted that it was not easy to test whether the students had achieved the learning outcomes or not. This kind of knowledge calls for alternative examinations and is not suited for traditional written exams.

“It is not declarative knowledge, such as reading a book chapter and then answering a written exam, but this is something that develops over time, encountering different situations and patients. So we have … we work a lot with reflective practice.” (Programme director of physiotherapy programme).

Since the implementation is ongoing and there is no full class that has yet gone through a national study programme with PCC implemented, no formal evaluations have been performed. The programme directors still shared that students seemed to appreciate learning content related to PCC, which is a facilitator. The fact that learning activities and suitable examinations were often time-consuming was identified as a barrier.

## Discussion

In our exploration of facilitators and barriers to implementation of PCC in Swedish national study programmes (first cycle) in medicine, nursing, occupational therapy and physiotherapy an overarching theme of PCC on the move became clear. PCC assumed different meanings and nuances in relation to existing syllabus content and pedagogical traditions at each educational setting. Although all programme directors described a movement towards what they interpreted as PCC, there was no consensus on what the end goal was and when PCC could be thought to be implemented ‘enough’.

Programme directors stated that PCC needed adaptation to the different local course syllabuses, and a number of understandings and levels of knowledge existed among them. An innovation having a vague definition is a previously known barrier towards implementation [30] and in a previous study reviewing local programme syllabuses and local course syllabuses we identified as many as 21 different terms connected to PCC [21]. A lack of clarity about what constitutes PCC was also present in a study of UK where teachers and leaders from nursing and medical programmes were interviewed [32]. Further, the complexity and different understandings of PCC were found in a recent interview study with HCPs practising PCC, where there were large variations in how they perceived it, for example, its value, the ethical underpinnings and how to operationalize it in practice [33]. The CFIR states both adaptability and complexity as essential factors for implementation, where a certain degree of adaptability is a facilitator for implementation [30]. However, to be able to adapt without losing the core components of the innovation these components need to be clearly defined [30]. The European standard for PCC [34] could be a starting point for the articulation of such core components. Even if all programme directors had stated that content relating to PCC, in some form, has been implemented, we cannot with this study design evaluate to what degree that is accurate. One could argue, however, that PCC has not been not implemented if the vocabulary is not used or if such vocabulary has just been tacked onto existing content.

From our findings it was also clear that not only was the concept itself vague, but also the teaching of PCC, that is, using the innovation was complex, and thus not all teachers might feel competent enough to take on the task. A study of higher education teachers’ experience of developing an interprofessional course on person-centredness showed that there were several challenges involved. There was a lack of familiarity with PCC among the teachers themselves and the educational activities they needed to master to be able to teach it [35]. A known barrier for implementation from the literature is a lack of competence in using the innovation [36]. We also found that programme directors experience that content related to PCC is not easy to grasp for students who need time and personal growth to master it. A study on student perspectives on learning PCC supports this and depicts learning PCC as an ‘uneasy journey’, concluding that self-reflection is needed to become a person-centred health care practitioner [37]. Moore et al. [32] suggest that to provide guidance relevant for an academic setting, the development of a ‘PCC skills competence framework’, particularly for teaching, is necessary. We would argue that such a framework could support higher education institutions to train and educate future HCPs for PCC. Moreover, an interprofessional PCC skills framework would also contribute to the greatest promise of PCC identified by the programme directors – namely, to form a joint language and an umbrella for all professions to gather under to work in teams. Thus, we suggest that not only a PCC skills competence framework on what a person-centred health care practitioner needs to master but also guidance for the teachers in didactic and pedagogical strategies on how to support students learning is necessary.

Even if PCC were perceived to have support in professional ethical codes, policy documents and law, its absence from national study programmes was identified as a barrier to implementation. In a UK setting, regulatory body requirements for nursing and medicine in favour of PCC were identified as key drivers for implementation in the national study programmes [32]. These findings are also supported by the CFIR, which points to the importance of external policies and incentives for implementation [30]. The programme directors in the current study, described a process of implementation carried forward mainly through informal processes and by individuals with a specific interest in PCC. Our previous study supports a finding that implementation of PCC is driven by bottom-up processes, as in initiated by local course leaders [21]. The programme directors described how the implementation was carried forward through champions such as professors and other influential persons who were positive towards and even had a research interest in PCC. Pratt-Chapman [38] found that implementation of new local course syllabus content relied on at least one strong higher education institution champion, someone who was passionate about the subject, and that such ‘in-house’ expertise was an important facilitator. Veer Ramjeawon et al. [39] also found that previous knowledge and experience of an innovation are important facilitators.

Known facilitators for implementation are, on the other hand, attending to systematic and well-organized implementation processes [40, 41] that have support from the organization [41, 42] and having sufficient planning, time and funding [40, 43, 44]. If the national study programme were to explicitly include PCC, it would probably provide incentives for a more formal, top-down implementation with sufficient resources set aside. However, informal implementation processes might be as efficient, while a forced, top-down implementation could create negative attitudes and lack of interest in PCC, which are both identified barriers in the literature [44, 45].

The present study identified the freedom of individual teachers and placement supervisors as a potential facilitator, if these were positive to PCC; otherwise, they could be a barrier. So, the freedom of teachers and placement supervisors could make them important champions for PCC. Other studies state that academic freedom, teamwork between actors within and outside the academy [35, 41], and their conviction about the innovation [42] are important facilitators for implementation. Moore et al. [32] also discussed academic freedom and reported on difficulties in ensuring that PCC policy was actually implemented in teaching in a consistent way. They discussed the possibility of students being exposed to ‘non-PCC practices’ at their clinical placements, which was an identified barrier also in our study. However, our results show that sometimes the sites for students’ clinical training had moved further towards PCC than the educational settings, which then facilitated implementation into the higher educational institutions.

## Conclusions

This study provides a deepened understanding of the current state of implementation of PCC in national study programmes, which seem to be an ongoing but ambiguous process moving at different paces. Programme directors express ambiguity and uncertainty around the meaning and value of PCC, and to what extent they are responsible for the implementation. More knowledge is needed about the core of PCC as a subject for teaching and learning – especially if it is to be able to function as a common language across professional boundaries. It is essential to strengthen teachers’ knowledge and skills on how teaching and learning PCC can be facilitated and provide guidance on sufficient pedagogical and didactic strategies to support students in becoming person-centred health care professionals.

## Limitations

To minimize limitations, we carefully addressed reflexivity throughout all phases of the research process [46, 47], that is, from the formulation of the research question to the drawing of conclusions. In relation to reflexivity we discussed the pros and cons of preunderstanding of the interviewers. All authors are affiliated to GPCC and stand clearly in favour of person-centred care. However, we considered it essential for the interviewer to have in-depth knowledge of person-centred care in order to bring forward rich interviews.

Trustworthiness is an overarching concept encompassing several aspects of qualitative studies, such as credibility, dependability, confirmability, transferability and authenticity [29]. Since it is the reader who judges the quality of a report [48], it is the authors’ responsibility to present the report in such a transparent way that the reader can evaluate its trustworthiness. We aimed to achieve transferability through providing citations and detailed information as well as examples from the data analysis. In terms of credibility, it is of significance to recruit participants who have knowledge of the phenomenon under study. Moreover, they should be suitable for interviews, meaning that they are willing to be interviewed and talk about the phenomenon. We chose programme directors, since they have the overall responsibility for the quality and content of educational programmes. However, the roles of programme directors differ between programmes and locations, and they are not always involved in the actual teaching. Even though there were, as a whole, rich descriptions of teaching and learning PCC, it is possible that teachers and course leaders would have presented other views on the implementation process. Using a theoretical framework like the CFIR for analysis could point attention to some parts of the data and thus away from other, possibly important, findings. We tried to avoid this by first using an inductive approach and being open to data, and then in a later phase using a deductive approach with the help of the CFIR.

## Supplementary Information

**Additional file 1.** Interview guide.

## Data Availability

The datasets analysed during the current study are available from the corresponding author on reasonable request.

## Abbreviations

CFIR: Consolidated Framework for Implementation Research
HCP: Health care professional
PCC: Person-centred care
