# Supplementary material for: Person-centred care on the move – an interview study with programme directors in Swedish higher education
Source: BMC Med Educ. 2022 Aug 1;22:589. doi: 10.1186/s12909-022-03657-4 (PMC9341055; doi:10.1186/s12909-022-03657-4)
Supplement: Supplementary file 1 — Additional file 1. Interview guide. [file 12909_2022_3657_MOESM1_ESM.docx]

# Interview guide

## Main questions

1. Have you or are you planning to implement person-centered care into your educational programme?
2. What concepts do you use in relation to person-centred care?
3. What are your experiences of how teaching and learning about person-centered care can be achieved?

## Examples of probe or follow up questions

- Would you like to elaborate on…?
- What do you mean by….?
- Can you give an example of….?
- What was the starting point for the implementation?
- Who were/are the driving forces in the implementation?
- Can you give me an example of something that was/is difficult regarding the implementation?
- What resources do you have at the university that you use for the implementation?
- What learning activities do you use in teaching about person/patient/client-centered care?
- How do the teachers experience teaching about person/patient/client-centered care?
- Has any specific training been given to the teachers?
